# Revisiting dezocine for opioid use disorder: A narrative review of its potential abuse liability

**DOI:** 10.1111/cns.70034

**Published:** 2024-09-18

**Authors:** Gordon A. Barr, Heath D. Schmidt, Ashish P. Thakrar, Henry R. Kranzler, Renyu Liu

**Affiliations:** ^1^ Department of Anesthesiology and Critical Care Medicine Children's Hospital of Philadelphia Philadelphia Pennsylvania USA; ^2^ Department of Anesthesiology and Critical Care Medicine, Perelman School of Medicine University of Pennsylvania Philadelphia Pennsylvania USA; ^3^ Department of Psychology University of Pennsylvania Philadelphia Pennsylvania USA; ^4^ Department of Biobehavioral Health Sciences, School of Nursing University of Pennsylvania Philadelphia Pennsylvania USA; ^5^ Department of Psychiatry, Perelman School of Medicine University of Pennsylvania Philadelphia Pennsylvania USA; ^6^ Division of General Internal Medicine, Department of Medicine, Perelman School of Medicine University of Pennsylvania Philadelphia Pennsylvania USA; ^7^ Penn Center for Addiction Medicine & Policy, Perelman School of Medicine University of Pennsylvania Philadelphia Pennsylvania USA; ^8^ Veterans Integrated Service Network 4 Mental Illness Research, Education and Clinical Center, Crescenz VAMC Philadelphia Pennsylvania USA; ^9^ Department of Neurology, Perelman School of Medicine University of Pennsylvania Philadelphia Pennsylvania USA

**Keywords:** biased mu‐opioid ligand, dependence, dezocine, drug discrimination, drug self‐administration, opioid use disorder

## Abstract

**Aims:**

Opioid use disorder (OUD) remains a serious public health problem. Opioid maintenance treatment is effective but under‐utilized, hard to access under existing federal regulations, and, once patients achieve OUD stability, challenging to discontinue. Fewer than 2% of persons with OUD stop using opioids completely. There have been calls from public advocacy groups, governmental agencies, and public health officials for new treatments for OUD. Dezocine, a non‐scheduled opioid previously used in the United States and currently widely prescribed in China for pain management, could be a candidate for a novel OUD treatment medication in the U.S. Nonetheless, to date, there have been no reviews of the clinical and preclinical literature detailing dezocine's abuse potential, a key consideration in assessing its clinical utility.

**Discussion:**

There are no English language reports of human abuse, dependence, or overdose of dezocine, despite years of extensive clinical use. There are a few case reports of dezocine abuse in the Chinese literature, but there are no reports of overdose deaths. Dezocine is perceived as an opioid and is “liked” by opioid‐experienced human and non‐human primates, properties that are not dose‐dependent and are mitigated by ceiling effects—higher doses do not result in more “liking.” There is little withdrawal, spontaneous or precipitated, in humans, monkeys, rats, or mice treated chronically with dezocine alone. However, at some doses, dezocine can precipitate withdrawal in humans and monkeys dependent on other opioids. In rodents, dezocine reduces the severity of morphine withdrawal and the rewarding properties of other opioids.

**Conclusions:**

Although dezocine is reinforcing in humans and monkeys with prior or concurrent opioid use within a restricted dose range, there are only a few anecdotal reports of dezocine abuse despite of the long history of use in humans. Given the evidence of dezocine's limited abuse potential, it could be useful both as a treatment for OUD. However, in‐depth studies would be required for dezocine to be re‐considered for clinical use.

## Graphical abstract

Dezocine is a partial MOR agonist previously used clinically in the USA and currently in China. It has been proposed that dezocine be reintroduced to clinical use in the USA, as an analgesia and/or an additional tool to treat opioid use disorder. To consider the clinical reintroduction of dezocine knowing its abuse liability is important. In this review, we found no reports of abuse, dependence or overdose in the English literature, and a small number of cases of dependence in the Chinese literature. If it is less or non‐addictive than other opioids, its short half‐life would allow it to bridge to other medication assisted therapies, facilitate tapering of opioid use and perhaps be used as a rescue medication in those trying to reduce opioid use.

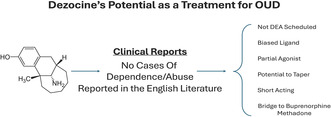

## 1 INTRODUCTION

Opioid misuse and dependence continue to be a public health crisis throughout the world.^1^ Opioid use disorder (OUD) remains a serious public health problem, affects over 35 million people worldwide, and more than 2.1 million in the United States alone. OUD mortality and morbidity have worsened with the spread of high‐potency synthetic opioids such as fentanyl in the unregulated drug supply. Leadership of the National Institute on Drug Abuse (NIDA) has called for new approaches and new medications.^2^, ^3^ We and others have argued for use of short‐acting opioids as an additional tool in the treatment of OUD.^4^, ^5^ Dezocine (Dalgan, WY‐16,255) is a short‐acting opioid with potent analgesic properties that was approved by the U.S. Food and Drug Administration (FDA) and used in the United States from1986 to 2011. Dezocine was not scheduled by the Drug Enforcement Administration (DEA). However, its abuse liability has not previously been reviewed, which is essential to considering it as a therapeutic drug.

### 1.1 Current medications for OUD

Maintenance therapy with methadone (first approved in 1972) and buprenorphine (first approved in 2002) is the cornerstone of effective OUD treatment.^6^, ^7^, ^8^ Evidence‐based treatment guidelines from the American Society of Addiction Medicine, the United States Department of Defense, the United Kingdom's National Health Service, the Australian Department of Health and Aged Care, and the Canadian Centre for Addiction and Mental Health, all recommend methadone and buprenorphine maintenance, given the long‐term safety and efficacy of these treatments for OUD. In the USA, Methadone for OUD treatment is available only from licensed opioid treatment programs. Buprenorphine can be prescribed for OUD treatment in any setting by a DEA‐registered provider. There is strong evidence that methadone and buprenorphine reduce all‐cause mortality, overdoses, use of non‐prescribed opioids, and infectious complications of injection drug use when patients remain in treatment for at least 6–12 months.^6^, ^8^, ^9^ Both drugs are opioid receptor agonists and are considered relatively safe and effective if used as prescribed; however, both have abuse potential and can be diverted. Considering whether dezocine has similar abuse liability is one goal of this review.

An ongoing problem in treating OUD is that there are low rates of enrollment in medication treatment—fewer than 20% of patients with OUD receive treatment with these medications each year. Furthermore, there are high rates of discontinuation of therapy—30%–70% of patients in treatment discontinue treatment in the first 6–12 months, particularly buprenorphine. Moreover, not all patients benefit therapeutically and maintenance with either is associated with use of other substances,^9^, ^10^, ^11^, ^12^ increasing the risk of fatal overdose, principally methadone. Moreover, transitioning patients from chronic opioid use to buprenorphine is limited because its μ‐opioid receptor (MOR) partial agonist activity can displace full opioid receptor agonists such as fentanyl from the MOR, making induction to treatment challenging. These advantages and challenges with current medications for OUD are summarized in Table 1.

**TABLE 1:** Status of medication‐assisted therapy For OUD.

Current status	Current problems
Methadone (1972), buprenorphine (2002) are FDA‐approved opioid receptor agonist treatments for opioid use disorder	Methadone and buprenorphine can be diverted or misused
Methadone and buprenorphine have strong evidence for reductions in all‐cause mortality, overdoses, use of non‐prescribed opioids, and infectious complications of injection drug use when patients remain in treatment for at least 6–12 months	Methadone is associated with overdose deaths, particularly during the first 4 weeks of treatment
Overdose fatalities with buprenorphine alone are uncommon	There is a high rate of discontinuation of both treatments with buprenorphine>methadone
Methadone for OUD treatment is only available from licensed opioid treatment programs	Not all patients benefit from methadone or buprenorphine and some individuals prefer treatment without opioid agonists
Buprenorphine for OUD treatment can be prescribed in any setting by a DEA‐registered provider	Both drugs are associated with weight gain with methadone>buprenorphine
	Transitioning a patient from chronic opioid use to buprenorphine is limited by the potential for the drug to displace full agonists from the mu‐opioid receptor, so induction to treatment with buprenorphine can be challenging.

### 1.2 A role for shorter‐acting opioids

One hallmark of these two drugs is that they are long‐lasting, from 24 h to 6 months for buprenorphine in current formulations, and 5–7 days terminal clearance for methadone. The long‐lasting actions serve to increase the effectiveness in treating OUD and reduce the likelihood of relapse. Yet it is the long half‐lives of these medications that limit the use of methadone and buprenorphine in situations where shorter‐acting opioid medications, such as dezocine, would be appropriate.^4^ The first such situation is to treat fentanyl withdrawal and help individuals using other opioids to initiate methadone or buprenorphine therapy,^13^, ^14^ two treatments that are effective in reducing patient‐directed discharge.^15^ Secondly, shorter‐acting opioid medications could be used for tapering off opioids should a patient wish to cease opioid use completely.^9^, ^10^, ^11^ Third, a shorter‐acting opioid medication could be used as a “rescue” strategy to reduce the cravings that promote relapse. These uses will be elaborated on in the “*Potential clinical uses of dezocine*” section below.

Dezocine (Dalgan, WY‐16,255) was approved by the U.S FDA in 1986. Its use was discontinued in the United States in 2011, for undisclosed reasons. It is a potent analgesic (clinically used at 0.10, 0.15 mg/kg). It is not a controlled substance and has been used extensively in China since 2009, for perioperative and cancer pain management, and it now represents approximately 45% of the opioid analgesic market in China.^16^, ^17^, ^18^

## 2 DEZOCINE'S MOLECULAR MECHANISMS

**FIGURE 1:**
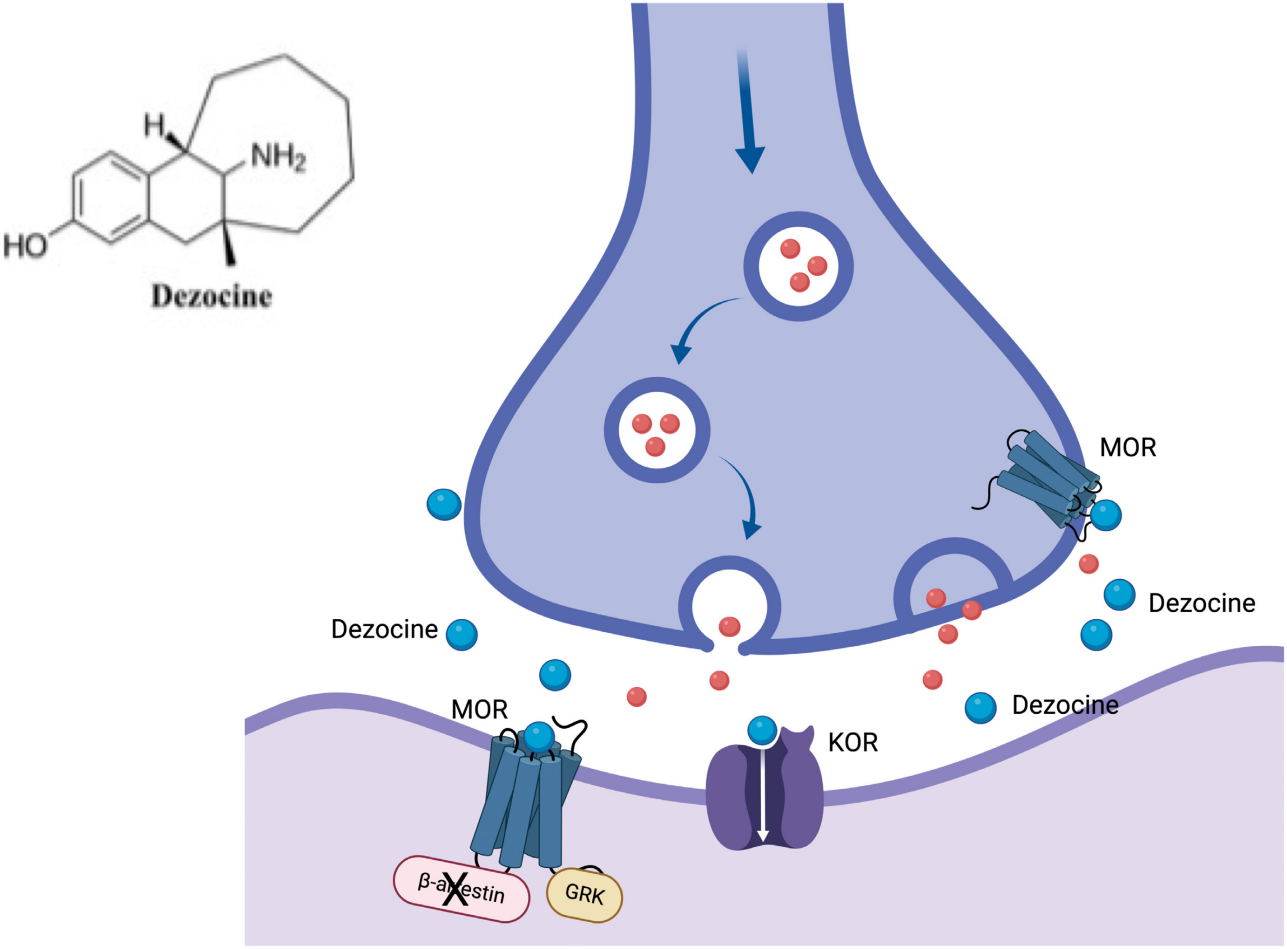
A schematic of dezocine's effects on opioid receptors. Dezocine is a MOR partial biased agonist without β‐arrestin activity and is a KOR mixed agonist/antagonist. Not shown—In vitro studies suggest that dezocine has norepinephrine uptake inhibitory properties at NE terminals. Created with BIoRender.com.

Dezocine's complex pharmacology as a biased partial μ‐opioid receptor agonist (MOR) may contribute to its unique physiological actions, leading to the argument that it should be repurposed as a treatment for OUD.^16^, ^19^, ^20^ Several prior comprehensive reviews have focused on the molecular mechanisms of action of dezocine^18^, ^19^, ^21^ and here we only summarize those mechanisms (See Figure 1).

### 2.1 Receptor binding at opioid receptors

Dezocine binds to opioid receptors with different affinities and intrinsic activities. It binds with high affinity to the μ‐opioid receptor  and κ‐opioid receptor (KOR) but binds weakly to the δ‐receptor (DOR).^19^, ^22^, ^23^, ^24^, ^25^ Specific affinities to humanized receptors in transfected cell lines are 3.7 ± 0.7 nM (MOR), 31.9 ± 1.9 nM (KOR) and 527 ± 70 nM (DOR).^19^ Dezocine docks to the same binding site as do endogenous ligands at both MOR and KOR.^19^ As a partial MOR agonist and a mixed KOR agonist/antagonist dezocine reduces the internalization of opioid receptors.^20^, ^26^

Dezocine's KOR antagonist activity was originally confirmed by functional ^35^[S]GTPγS binding. Dezocine alone did not stimulate KOR‐mediated G‐protein activation but inhibited the full κ agonists nalbuphine and salvinorin A‐induced G‐protein activation, resulting in the classification as a KOR antagonist.^19^, ^24^ In some assays, dezocine was a mixed KOR agonist/antagonist (O'Brien and Benfield, 1989; Wang et al., 2018). Dezocine was a partial agonist at both the KOR and MOR, significantly reducing the G‐protein stimulation by the full KOR agonist U50,488H and the full MOR agonist DAMGO.^25^ In behavioral assays, dezocine was antinociceptive in an inflammatory pain assay, with effects that were antagonized by the KOR antagonist nor‐BNI and MOR antagonist β‐FNA, consistent with dezocine being a KOR and MOR agonist. But when dezocine was administered with U50,488H or morphine, it reduced the antinociceptive effects of both drugs, suggesting that it also has antagonist activity.^25^

### 2.2 Biased signaling

The two intracellular signaling paths, G‐protein and β‐arrestin, mediate different functions. Dezocine is a G‐protein agonist at MOR. Alone, it stimulated G‐protein activation in cells expressing κ and μ receptors; in the presence of full KOR agonist U50,488H and μ agonist DAMGO, dezocine inhibited U50,488H‐ and DAMGO‐mediated G‐protein activation. Although initial reports suggested there was no significant G‐protein activation with dezocine at KOR, indicating a lack of agonist activity, when the cells were activated with full agonists (nalbuphine or salvinorin A), dezocine inhibited the agonist concentration‐dependently suggesting full receptor antagonist activity.^19^

There has been recent interest in developing biased opioid receptor agonists that selectively target G‐protein signaling over β‐arrestin signaling, initially hoping that this would reduce side effects and addiction liability.^27^, ^28^ This initial hope has turned out to be more complicated.^28^ Morphine and buprenorphine induce β‐arrestin recruitment, in particular, buprenorphine. Dezocine in contrast does not induce the β ‐arrestin path.^29^ The absence of β‐arrestin activity may account for some of its unique pharmacological properties.

### 2.3 NET and SERT

In addition to opioid receptors, dezocine targets two novel pharmacological sites—the norepinephrine (NET) and serotonin (SERT) transporters. Dezocine is predicted to share the same binding site with nisoxetine, a specific NET inhibitor, at the NET site. Dezocine also shares the same binding site for desipramine.^19^ Both findings indicate that dezocine may share the same site as selective serotonin reuptake inhibitors or tricyclic antidepressants. Competitive binding studies, however, complicate this story. Dezocine inhibited the norepinephrine transporter but less potently than did the selective NET inhibitor nisoxetine and like nisoxetine dezocine had inhibitory actions only at high concentrations at the SERT. Whether this monoamine reuptake inhibition and potential antidepressant properties of dezocine are functional is not known, but there is both clinical and animal work that suggests that they may.^30^, ^31^, ^32^ These data are reviewed in the section on antidepressant actions below.

There always should remain concerns about the abuse potential of all opioid drugs. Dezocine may have a reduced potential for abuse, but studies with humans and non‐human primates suggest that the risk is not zero. Below we review the data on the potential abuse and reinforcing properties of dezocine. Studies including females are rare and in those instances the reports do not address sex differences, and thus, despite their importance, sex differences are not considered here.

## 3 SCOPE OF THIS REVIEW

Dezocine has multiple properties that have been actively studied. These include its analgesic profile alone^18^ or in combination with drugs such as dexmedetomidine^33^, ^34^ and other opioids such as sufentanil,^35^, ^36^ and its ability to stimulate immune function^18^, ^33^, ^37^, ^38^ with potential anti‐tumorigenic properties.^39^, ^40^, ^41^ These properties of dezocine, although important, are beyond the scope of this review which focuses the potential abuse risk and its potential for treating OUD.

## 4 CLINICAL REPORTS‐ENGLISH LANGUAGE

To our knowledge, at the time of this review, there are no published reports of addiction, dependence, or overdose with dezocine in the English language literature. Thorough searches using Medline or ClinicalTrials.gov or of the wider literature with the artificial intelligence‐based search engines System Pro, LitMaps and Research Rabbit failed to turn‐up a single report of abuse/overdose despite our use of multiple search term combinations. Dezocine's lack of abuse liability may reflect its perioperative parenteral use, making it less accessible to diversion than other drugs. However, drugs used in similar ways, such as propofol, are abused.^42^ Thus, the use of dezocine, which is largely restricted to the operating room, cannot fully explain its lack of abuse reports in the literature. Dezocine is now approved in China for cancer pain management, which may provide additional data on its potential for addiction following chronic use.

On February 27, 1992, Astra—the pharmaceutical company that marketed dezocine (Dalgan)—reported to the FDA's Drug Abuse Advisory Committee that after 2 years on the market, approximately 0.2% of the “over half a million” units shipped were unaccounted for and postulated that some of this loss may have been due to abuse (*Source*: Astra VP‐Medical Affairs Nigel Rulewski, reported in *The Pink Sheet, March 2, 1992*). It was further noted that the only potential abuse suspected by Astra were two doctors who repeatedly reordered dezocine, claiming that shipments were missing or incomplete. The summary of the meeting did not detail the total number of physicians ordering dezocine, so it is unclear what percentage of physicians those two represent. Astra surveyed 198 hospitals that purchased dezocine directly, which included a total of 721 physicians, 82% of whom considered dezocine a prescription medication, not a controlled substance. None had any knowledge of diversion, theft, or abuse of Dalgan. There also appeared to be an expectation that Astra would follow up with surveys of NIDA‐approved treatment centers regarding abuse of Dalgan, but we could find no evidence as to whether those surveys were conducted.

The FDA Adverse Events Reporting System (FAERS) maintains records of adverse events reported by health care professionals or consumers. During the time that dezocine was on the market (1986–2011) a total of 103 adverse events were reported (note—a single case can have multiple events). The reported adverse events range widely and included ineffectiveness, medication error, vasodilation, injection site pain, and nausea/vomiting. Most relevant here is the “Drug Dependence” event, as defined by the MedDRA dictionary, which was listed in 38 of the 103 events. It is difficult to assess the importance of these 38 cases over 25 years as little detail is available about each. However, to provide perspective, during the same 25‐year period, there were approximately 17,000 adverse event reports for propofol and close to 6000 for morphine. Rates for comparison across drugs is not possible, however, as the denominator (the number of prescriptions for these drugs) is unavailable.

## 5 CLINICAL REPORTS‐CHINESE LANGUAGE

Searches of the Chinese literature using the Wanfang Medical Database (a database similar to PubMed), returned a compendium of eight cases from the literature.^43^ All cases in that compendium review were also identified by searches of PubMed, and the Wanfang Medical Database and additionally by searching the China National Knowledge Infrastructure (CNKI). None of the cases were in the foreign databases; all were found in the Chinese literature. We also identified a case using Wanfang searches published after the compendium review was published.^44^ Of the eight cases, seven were young to middle‐aged adults, five men, three women and use typically started as treatment for pain (surgery, injury, dysmenorrhea, cancer). In two cases the dosage was higher than the maximum recommended therapeutic dosage (120 mg/day) whereas the other six were not. Dependence was not dose‐dependent but occurred rapidly within 30 days in five cases. The duration of dependence was less than 60 days in four cases and more than 1 year in four cases. Of the 8 cases, one was lost to follow‐up, one was in progress and six were “cured.” Although details of the “cures” were not provided, the authors state that withdrawal treatment was relatively simple and relatively brief.^43^

The later case was of a person living with HIV who was seen in the author's hospital. The patient was treated with dezocine continuously for over 40 days to treat pain from hand injuries. Although dependence was not formally assessed, the patient's irritability and increased pain upon reduction of the dezocine dosage led the clinicians to believe he was dependent. Treatment of the dependence included the cessation of dezocine treatment, treatment with sedatives and hypnotics, and a psychological intervention. Within 4 days, the withdrawal signs dissipated and the patient was discharged.^44^

In summary, although there are no English language reports of addiction/dependence with dezocine there are a handful of reports in the Chinese literature, and it is generally accepted there that dependence on dezocine can occur. However, given the low number of cases, the risk is likely to be low in view of the widespread use of dezocine in China. Moreover, we found no cases of clearly identified OUD. Dependence on dezocine was apparently easily treated. Of the eight cases, only one case involved combining dezocine other drugs of abuse. This is in contrast to the high incidence of polydrug abuse, including other opioids, stimulants, and benzodiazepine sedatives seen clinically in China [45 cited in 43]. Thus, overall, it is unlikely that dezocine has zero risk of dependence, but rather a lower risk of dependence than other opioids analgesics.

## 6 EXPERIMENTAL STUDIES

### 6.1 Drug subjective and discrimination effects

#### 6.1.1 Subjective effects

The subjective effects of dezocine in humans irrespective of prior opioid exposure are complex.^46^, ^47^, ^48^ Subjects with a history of opioid use but no current use or dependence recognized dezocine (15, 30 or 60 mg per subject) as an opioid, like morphine, and universally liked it.^46^ Dezocine increased Euphoria ratings on the Addiction Research Center Inventory (ARCI; Morphine‐Benzedrine Group (MBG)‐subscale) instrument at the two highest doses, which did not differ from one another. Thus, there was no increase in euphoria scores when the dose was doubled from 30 to 60 mg,^46^ suggesting a ceiling effect (i.e., higher doses did not result in increased “euphoria”) as there is with its respiratory depressive actions [i.e., higher doses did not result in more respiratory depression^48^, ^49^]. Scores for “dysphoria” on another scale of the same ARCI instrument [Lysergic acid diethylamide Specific Group (LSD)‐subscale] did not differ from placebo. Volunteer participants, with a limited history of opioid use, but not abuse, liked dezocine and described the effects as “good mood, drunk, coasting, and happy” equally at all doses (2.5–10.5 mg/70 kg), but also reported increased “dysphoria” (LSD‐scale) at the same time but now in a dose‐dependent manner.^47^ This replicates prior work in which healthy volunteers with no opioid history who received dezocine (10–40 mg/70 kg) “did not find the effects of dezocine pleasant”, especially compared to morphine. The unpleasant rating persisted with cumulative dosing and was particularly strong at the higher doses.^48^ Thus, positive feelings occurred over a wide range of doses but were limited by a ceiling effect combined with increased dysphoria starting at lower doses. Together, these responses may explain dezocine's limited abuse liability as evidenced by the lack of reported cases of abuse.

#### 6.1.2 Self‐administration

In an intravenous (i.v.) drug self‐administration paradigm, dezocine substituted for codeine in macaque monkeys. The dezocine dose–response curve (0.01–0.30 mg/kg/infusion) was shallow (i.e., the differences among doses were small), and dezocine was less potent than were most other MOR agonists tested. Response rates in the operant task were far lower for dezocine than for other opioids tested, except nalbuphine, implying that the monkeys were less willing to work for dezocine than for most MOR agonists. Moreover, the only effective dose was 0.1 mg/kg/infusion; increasing the dose (0.30 mg/kg/infusion) slightly reduced the response rate.^50^ Thus, like other opioids, dezocine was self‐administered by an already dependent animal, although in a complex manner, again with a ceiling effect. Whether dezocine would be self‐administered in a drug‐naïve subject has not, to our knowledge, been tested. If dezocine were to be used in an OUD population, and if it is not reinforcing and self‐administered in naïve subjects, it would have a reduced risk of abuse by diversion to non‐OUD persons.

#### 6.1.3 Drug discrimination

In macaque and squirrel monkeys, dezocine generalized to morphine or etorphine in a drug discrimination task in some,^50^, ^51^ but not all, studies.^17^ These differences cannot be explained by differences in dosage as all three studies tested similar dose ranges. In one non‐mammalian report, pigeons discriminated dezocine from saline, and the μ‐opioid receptor agonists fentanyl or morphine substituted fully for dezocine.^52^ The ability of monkeys to discriminate all three agonists, morphine, fentanyl, and dezocine, from vehicle was reversed by naloxone. However, there were differences: when compared to the ability of naloxone to block either fentanyl/saline or morphine/saline discriminations, dezocine required a 10‐fold higher dose to block the pigeon's ability to discriminate dezocine from the vehicle comparator. κ‐Opioid receptor agonists did not substitute for dezocine, suggesting that dezocine's subjective effects are not dependent on activity at the κ‐opioid receptor.^52^

#### 6.1.4 Withdrawal

When methadone‐dependent men were injected with dezocine (7.5, 15, 30, 45, 60 mg/per subject), it precipitated dose‐dependent withdrawal at the middle dose (30 mg) and to a lesser degree, the 45‐mg dose. On a visual analog scale, the men described dezocine's effects at that dose as “bad”, feeling “sick” and as “withdrawal.” There was no “high” or “liking” the drug. Independent experimental observers also noted withdrawal signs (Himmelsbach Opioid Withdrawal Scale, “Total Score”) but again only at the 30‐ and 45‐mg doses, confirming the self‐reports. The individual withdrawal signs on that scale were limited to yawning and restlessness.^53^ Neither the lower two doses nor the highest dose produced withdrawal. Subjectively, the lower two doses were generally rated as placebo whereas the middle dose (30 mg) was reported mostly as an antagonist. The 45‐mg dose was identified as a placebo, antagonist, and agonist by similar numbers of subjects whereas the highest dose (60 mg) was labeled as placebo or an agonist in their ratings. Thus, as with the euphoric effects, dezocine's ability to precipitate withdrawal is limited to a narrow dose range, which suggests that doses could be titrated to avoid precipitating withdrawal.

Consistent with the human data, dezocine acted as an antagonist in morphine‐dependent macaques, precipitating withdrawal [0.5, 1.0 mg/kg^17^]. In mice, dezocine did not reduce the “jumping” that accompanies precipitated withdrawal, but in rats dezocine reversed morphine‐induced loss of righting over a wide range of dezocine doses (0.5–10 mg/kg),^17^ and dezocine reduced naloxone‐precipitated withdrawal in opioid‐dependent mice and rats.^20^, ^30^ Yet, following repeated dosing, dezocine itself does not induce physical dependence in humans, monkeys, rats, or mice, as evidenced by no spontaneous, and little opioid‐antagonist‐precipitated withdrawal.^17^, ^20^, ^30^ This is true, even with continued hourly intravenous dezocine infusions in macaques for up to 30 days [0.50 mg/kg/infusion^17^]. In addition, high doses of dezocine did not produce visible behavioral signs, withdrawal signs, or toxic effects in monkeys at doses much higher than the clinically used analgesic doses [0.10–0.15 mg/kg^17^, ^18^, ^49^].

### 6.2 Effects in rodent models of reinforcement

Dezocine blocks many of the rewarding and reinforcing effects of opioids in rodent models of OUD. In the conditioned place preference (CPP) paradigm, when the preference for a morphine‐paired environment is extinguished, a single dose of morphine will “reinstate” that preference with no additional training. Dezocine (1.25 mg/kg) blocked the reinstatement of CPP elicited by morphine, suggesting that it reduces/abolishes morphine's rewarding cues.^20^

Because dezocine has such a low pH, it must be injected i.v. or i.m. To circumvent this limitation, a recently developed and patented dezocine‐cyclodextrin complex at a neutral pH termed Cyc‐dezocine was developed to be given intranasally to avoid the need for injection [Cyc‐dezocine; also termed nano‐dezocine^29^]. Cyc‐dezocine can penetrate the blood–brain barrier. Cyc‐dezocine given systemically or intranasally reduced oxycodone self‐administration in two distinct paradigms. When rats self‐administered oxycodone either by i.v. infusion in an operant task or by drinking oxycodone in a 24/7 full‐access two‐bottle model, Cyc‐dezocine significantly reduced the voluntary consumption of oxycodone.^54^ Rats in the i.v. self‐administration paradigm were maintained on a fixed‐ratio schedule of reinforcement where every 5 lever presses (FR‐5) resulted in an i.v. infusion of oxycodone. The 10 mg/kg intraperitoneal (i.p.) dose was effective in reducing oxycodone self‐administration, with 5 mg/kg showing a trend toward decreased consumption. In the second paradigm, female and male rats had full 24/7 access to both a water bottle and a bottle containing oxycodone. Rats quickly develop a strong preference for drinking oxycodone, averaging ~10 mg/kg/day. After about 6 weeks of voluntary intake, rats were injected intranasally with 2 mg/kg of Cyc‐dezocine and placed back in their home cages. On average, rats given Cyc‐dezocine drank ~2.6 times less oxycodone than those treated with vehicle. Thus, the combined results of Cyc‐dezocine in the two self‐administration paradigms and of dezocine in the CPP reinstatement paradigm suggest that dezocine reduces the rewarding and reinforcing properties of opioids in rats with a history of, or concurrent use of, opioids.

### 6.3 Antidepressant actions

In a study of postoperative depression, which is common among patients undergoing cancer surgery, patients who received dezocine in addition to a pain medication had lower scores on the Beck Depression Inventory than did patients who received the pain medication alone.^32^ In mouse models of “depressive‐like” behaviors—the forced swim task and tail suspension task—immobility is used as a proxy for the clinical state. In both tasks, dezocine dose‐dependently reduced immobility, with no other motor effects. The reduction in immobility was blocked by the serotonin 1A receptor antagonist Way100635 and by the κ‐opioid receptor agonist U50,488, consistent with dezocine acting via serotonin and κ‐opioid receptors.^31^ Although dezocine itself produces minimal precipitated withdrawal after chronic treatment, withdrawal signs were partially evident after yohimbine, and α2 adrenergic receptor antagonist, treatment suggesting that the potential dependence‐producing properties of dezocine could be constitutively reduced by increased noradrenergic activity at the α_2_ noradrenergic receptor.^30^ Given that depression often co‐occurs with OUD,^55^, ^56^ these pharmacological effects of dezocine could be beneficial in treating OUD and may contribute to reductions in morphine CPP and/or oxycodone self‐administration following dezocine administration.

## 7 POTENTIAL CLINICAL USES OF DEZOCINE

As others have argued,^16^ it may be time to reconsider dezocine's role in pain management, OUD treatment, or both. Although it is the long half‐lives of methadone and buprenorphine that contribute to their clinical efficacy, these long half‐lives also limit their use in situations where a shorter‐acting opioid medication would be more efficacious. We envisage at least three clinically important uses of dezocine. The first is to treat fentanyl withdrawal and help individuals using fentanyl initiate methadone or buprenorphine treatment. Short‐acting potent opioids that rapidly mitigate symptoms of pain and withdrawal, especially in hospital settings, can serve as a bridge to long‐acting methadone or buprenorphine therapy on discharge.^13^, ^14^ This would allow patients at risk to experience less withdrawal and continue their treatment. Such treatment of withdrawal and pain by other short‐acting opioids reduced early patient‐directed discharge and increased medically advised stay.^15^ Dezocine is as efficacious and equipotent to morphine in pain relief with an apparent ceiling effect on both its respiratory depressant actions^49^ and its opioid‐like subjective effects^46^, ^47^ making it a viable option for this short‐term use.

The second use for dezocine would be to help people with OUD taper off opioids.^4^ Current maintenance therapies will continue to have a significant role in treating OUD since they are safe, effective, and reduce all‐cause mortality and morbidity across a range of outcomes. However, there is a subset of patients with OUD who prefer treatment without opioid receptor agonists and who need help reducing cravings and withdrawal without transitioning to long‐term maintenance on buprenorphine or methadone. There are also patients maintained on buprenorphine or methadone who reach stability in their OUD treatment and who wish to taper off opioid receptor agonists. Patients who discontinue methadone treatment for OUD do so for multiple reasons^57^ but are at increased risk of overdose and death once they stop the maintenance treatment.^58^, ^59^ This subset of patients might benefit initially from gradual opioid tapering using quick‐onset and short‐acting opioids that have low addiction potential. Because both methadone and buprenorphine have long half‐lives, tapering is difficult. Short‐acting opioids would ease the process of tapering opioid use like the taper‐down strategy used to discontinue high dose corticosteroid treatment. Due to its relatively short half‐life, dezocine tapering might require less time than longer‐acting treatments. The complex dysregulation of the endogenous opioid system created by chronic opioid exposure would slowly return to its normally regulated state. The goal would not be to use dezocine as a replacement therapy, but rather as an adjunct to facilitate the gradual weaning from opioids.

Third, and related to the second use, dezocine could be used as a “rescue” medication to reduce the cravings that promote relapse. Relapse rates are high early in treatment with methadone and buprenorphine. Animal studies show that dezocine reduces the reinstatement of morphine‐induced conditioned place preference after extinction.^20^ Clinically, the strategy of using a long‐acting agonist in conjunction with an as‐needed short‐acting agonist is effective in reducing smoking relapse with the addition of gum or lozenges to nicotine patches.^60^, ^61^ A similar strategy could be effective for OUD and could be tested empirically.

## 8 FUTURE DIRECTIONS

Given the data reviewed above, dezocine appears to have less potential for abuse than do other opioids. Yet the data are sparse. There are several directions that are required to characterize further dezocine's abuse liability and potential as a short‐acting opioid to supplement current long‐lasting maintenance therapies such as methadone and buprenorphine. For example, would dezocine be self‐administered by drug‐naïve subjects? Individuals with OUD appear to like it but non‐opioid using individuals appear to not. This is important because if dezocine is not self‐administered in drug‐naïve subjects, there could be less diversion risk when treating individuals with OUD. Second, there are virtually no data on sex differences in the literature, with most data on male subjects, both human and non‐human. Given sex differences in OUD risk, treatment outcomes, and psychiatric co‐morbidities,^62^, ^63^, ^64^, ^65^ future studies need to include both sexes. Finally, due to its low pH, dezocine is administered i.v. or intramuscularly clinically. The new formulation, Cyc‐dezocine, because of its neutral pH and intranasal route of administration with good penetration to the brain,^29^ shows promise as it reduced oxycodone self‐administration in two different paradigms.^54^ Whether either dezocine or Cyc‐dezocine would be effective in the treatment of OUD is an important, but open, question.

## 9 CONCLUSIONS

In summary, both the lack of reports of misuse of dezocine and the preclinical literature support the possibility that dezocine has less abuse potential than do other opioid analgesics. Dezocine is perceived as an opioid and “liked” by opioid users, but the drug‐liking appears to be based on the extent of prior opioid exposure. Higher doses do not uniformly result in increased liking and may increase disliking of the drug. In non‐human primates, dezocine substitutes for other opioids and for codeine, but within a restricted dose range and at lower rates than other opioids. Dezocine can precipitate withdrawal among opioid‐dependent humans and monkeys. This opioid receptor antagonist property might limit the abuse potential of dezocine.^46^ We identified no studies in which dezocine was self‐administered by drug‐naïve individuals. However, in all species tested, including humans, cessation of treatment following repeated administration of dezocine did not result in spontaneous withdrawal; nor were there major withdrawal symptoms precipitated by administration of an opioid receptor antagonist. In rodents, dezocine reduces precipitated withdrawal, blocks morphine‐induced reinstatement of CPP, and reduces oxycodone self‐administration. Dezocine may have antidepressant activity that could be helpful in treating persons with OUD. Finally, dezocine is currently not a scheduled drug, consistent with its greater safety profile than either methadone or buprenorphine—particularly with respect to overdose risk. Thus, dezocine could fill an important gap in the medication‐assisted treatment of OUD, specifically when use of short‐acting opioids is appropriate.

## AUTHOR CONTRIBUTIONS

Conceptualization: GAB, HDS, RYL. Roles/Writing—original draft and revisions: GAB. Writing—additional content, review, critique & edit: HDS, APT, HRK, RYL.

## FUNDING INFORMATION

Dr. Liu acknowledges the funding support from NIH (R01:1R01GM111421). Dr. Schmidt is supported by the following grants from NIH: R01 DA037897 and R21 DA057458. Dr. Thakrar is supported by NIH grant R34 DA057507. None of these three grants provided funds used for the preparation of this manuscript. No other funding for this work is declared. No generative AI or any other AI‐assisted technologies were used in the writing process.

## CONFLICT OF INTEREST STATEMENT

Dr. Barr is the Director of Medicinal Development at NeuroKappa Therapeutics LLC but receives no compensation from it. Cyc‐dezocine is one of its products. Dr. Schmidt has no competing financial interests. Dr. Thakrar has no competing financial interests. Dr. Kranzler is a member of advisory boards for Altimmune, Dicerna Pharmaceuticals, Sophrosyne Pharmaceuticals, Enthion Pharmaceuticals, and Clearmind Medicine; a consultant to Sobrera Pharmaceuticals; the recipient of research funding and medication supplies for an investigator‐initiated study from Alkermes; a member of the American Society of Clinical Psychopharmacology's Alcohol Clinical Trials Initiative, which was supported in the last 3 years by Alkermes, Dicerna, Ethypharm, Lundbeck, Mitsubishi, Otsuka, and Pear Therapeutics; and a holder of U.S. patent 10,900,082 titled: “Genotype‐guided dosing of opioid agonists,” issued 26 January 2021. Dr. Liu is founder of NeuroKappa Therapeutics LLC and the lead inventor for the U.S. patent (Priority to US15/967,526), titled: Compositions and methods for treating opioid receptor‐associated diseases; issued in 2018. The University of Pennsylvania is the patent holder. Dr. Liu receives no compensation from the company.

## Data Availability

Data sharing not applicable to this article as no datasets were generated or analysed during the current study.
